# A novel plasmid‐based experimental system in *Saccharomyces cerevisiae* that enables the introduction of 10 different plasmids into cells

**DOI:** 10.1002/2211-5463.13893

**Published:** 2024-10-10

**Authors:** Geyao Dong, Tsuyoshi Nakai, Tetsuo Matsuzaki

**Affiliations:** ^1^ Department of Neuropsychopharmacology and Hospital Pharmacy Nagoya University Graduate School of Medicine Japan; ^2^ Department of Pharmacotherapeutics and Informatics Fujita Health University School of Medicine Toyoake Japan

**Keywords:** auxotrophy, plasmid, protein expression, *Saccharomyces cerevisiae*

## Abstract

The budding yeast *Saccharomyces cerevisiae* is commonly used as an expression platform for the production of valuable compounds. Yeast‐based genetic research can uniquely utilize auxotrophy in transformant selection: auxotrophic complementation by an auxotrophic marker gene on exogenous DNA (such as plasmids). However, the number of required auxotrophic nutrients restricts the number of plasmids maintained by the cells. We, therefore, developed novel Δ10 strains that are auxotrophic for 10 different nutrients and new plasmids with two multiple cloning sites and auxotrophic markers for use in Δ10 strains. We confirmed that Δ10 strains were able to maintain 10 types of plasmids. Using plasmids encoding model proteins, we detected the co‐expression of 17 different genes in Δ10 cell lines. We also constructed Δ9 strains that exhibited auxotrophy for nine nutrients and increased growth compared to Δ10. This study opens a new avenue for the co‐expression of a large number of genes in eukaryotic cells.

## Graphical abstract

We developed the *Saccharomyces cerevisiae* strains termed Δ10 that exhibit auxotrophic behavior for 10 different nutrients and the plasmids with auxotrophic markers for use in Δ10 strains. Δ10 strains were able to maintain 10 types of plasmids by auxotrophic selection. This Δ10‐based experimental system enables co‐expression of a large number of genes, which is valuable for both basic and applied science.

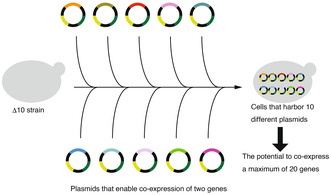

**Abbreviations**
SD: synthetic dextrose
SG: synthetic galactose
YPAD: yeast extract‐peptone‐adenine‐dextrose

The budding yeast *Saccharomyces cerevisiae* is one of the most widely used eukaryotic model organisms in laboratory experiments because of the ease and precision with which its genome can be manipulated, and the high degree of conservation of cellular processes and molecular pathways in higher eukaryotes, including humans [1, 2, 3, 4]. In addition to being an important model organism for basic studies, *S. cerevisiae* is also an invaluable tool in the field of biotechnology [5, 6]. Recent developments in synthetic biology have facilitated the engineering of yeast to produce fuels, food ingredients, and biopharmaceuticals [7, 8, 9, 10, 11, 12, 13, 14, 15, 16, 17, 18].

An important aspect of yeast genetics is the use of multiple auxotrophic yeast strains that carry mutations in genes involved in biosynthetic pathways, typically those essential for amino acid or nucleotide biosynthesis [19, 20]. For example, *URA3* encodes orotidine‐5′‐phosphate decarboxylase, an essential enzyme in the *de novo* biosynthesis of pyrimidine [21]. *URA3* null mutants are defective in uracil biosynthesis and, therefore, cannot grow on media lacking uracil. Transformants containing exogenous DNA (such as plasmids) encoding wild‐type *URA3* were selected as viable clones in media lacking uracil. Thus, *URA*3 was used as an auxotrophic marker for *URA3* null mutants. Similarly, *HIS3*, *LEU2*, *TRP1*, *LYS2*, *MET15*, and *ADE2* encode enzymes essential for l‐histidine, l‐leucine, l‐tryptophan, l‐lysine, l‐methionine, and adenine biosynthesis, respectively, and are often used as auxotrophic markers [19, 20].

W303 and BY4741/BY4742 are two widely used laboratory strains [19, 20]. W303 carries mutations in *URA3*, *ADE2*, *TRP1*, *LEU2*, and *HIS3*, which are frequently employed as auxotrophic markers. BY strains are wild‐type for *TRP1* and *ADE2*, but BY4741 and BY4742 are deficient in *MET15* and *LYS2*, respectively. The limited availability of auxotrophic markers restricts gene manipulation. This complicates efforts to engineer yeast to produce chemicals because a large number of heterologous genes must be introduced to reconstitute the biosynthetic pathways of the target products. For example, the total synthesis of opioids in yeast has been achieved by co‐expressing more than 20 heterologous genes [22].

In this study, we created novel Δ10 strains (YMT183–185) that carry null mutations in 10 different auxotrophic markers and a series of plasmids that encode a wild‐type allele of an auxotrophic marker deleted in Δ10 strains, which also contained two multiple cloning sites. We also confirmed that 10 plasmids could be introduced into Δ10 strains. Since two different genes can be co‐expressed from a single plasmid, this study may make it feasible to co‐express a maximum of 20 different genes. We also created Δ9 strains that exhibit auxotrophy for nine nutrients accompanied by faster growth compared to Δ10.

## Materials and methods

### Strains, plasmids, primers, and media

**Fig. 1:**
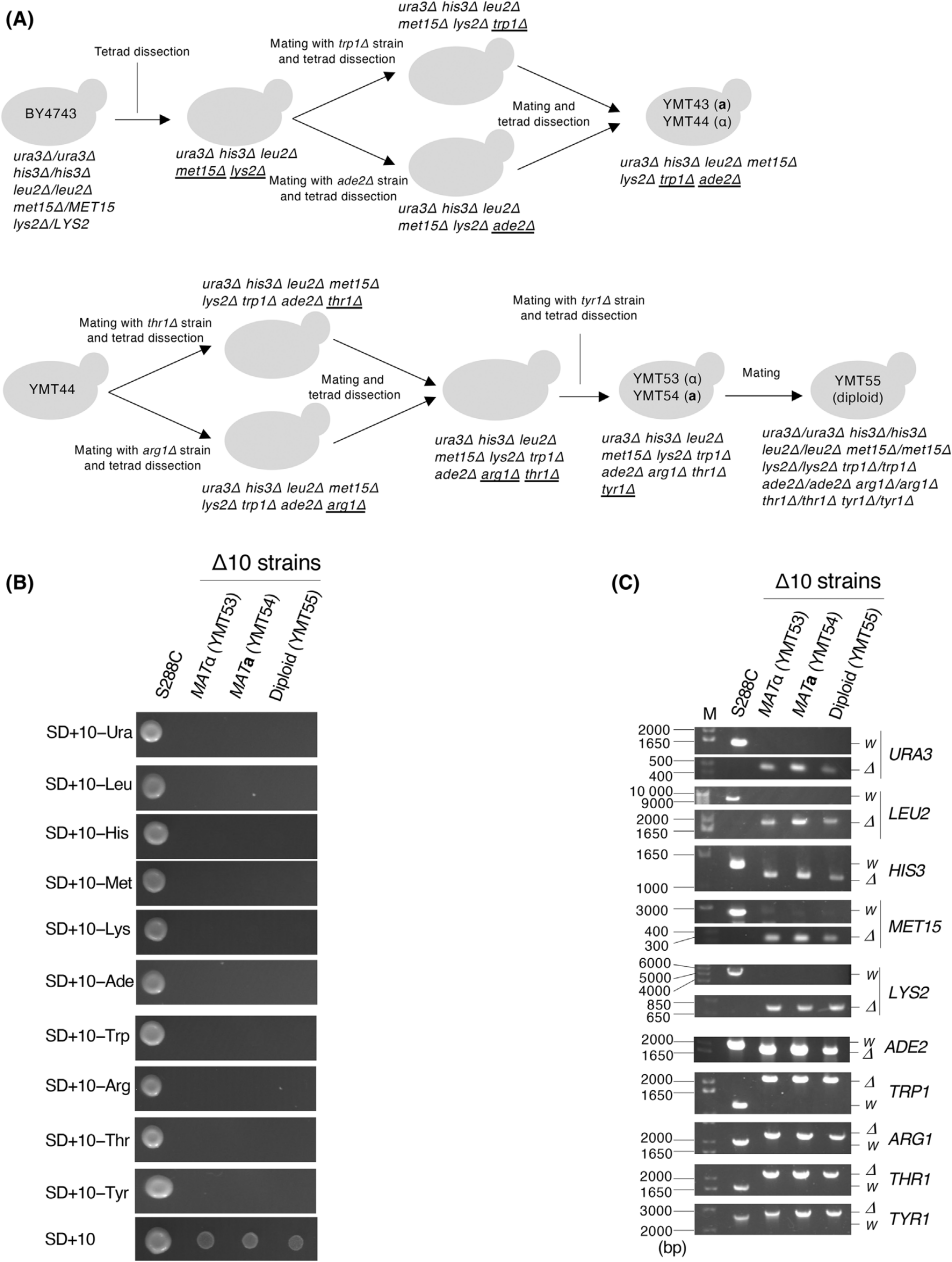
Construction of Δ10 strains. (A) Schematic procedure for construction of the Δ10 strains YMT53–YMT55. Underlined letters indicate the mutation introduced at each step. (B) Δ10 strains were auxotrophic for 10 different nutrients: uracil, leucine, histidine, methionine, lysine, adenine, tryptophan, arginine, threonine, and tyrosine. Cells were spotted and cultured on indicated plates for 2 days at 27 °C. The S288C strain, which does not exhibit any auxotrophic behavior and is the ancestor of BY4741/BY4742, was used as a control. (C) Deletion of each auxotrophic marker in Δ10 strains was confirmed by PCR. M, 10‐kbp DNA marker; *W*, wild‐type allele; *Δ*, deletion allele.

S288C, BY4741, BY4742, and BY4743 were purchased from Open Biosystems (Cambridge, UK). The *trp1Δ*, *ade2Δ*, *thr1Δ*, *arg1Δ*, and *tyr1Δ* strains, which were used to construct Δ10 strains YMT53–YMT55 (Fig. 1), were obtained from a collection of nonessential gene deletion strains purchased from Open Biosystems (Cambridge, UK). The plasmids pESC‐URA, pESC‐LEU, pESC‐HIS, and pESC‐TRP were purchased from Agilent Technologies (Santa Clara, CA, USA). Details of the strains, plasmids, primers, and media used in the study are described in Tables S1–S4, respectively.

### Construction of Δ10 strains

A schematic of the construction of Δ10 strains (YMT53–YMT55) is shown in Fig. 1A. First, BY4743 was sporulated and the resulting tetrads were dissected. Colonies that did not grow on media lacking either lysine or methionine (i.e., these colonies were auxotrophic for lysine and methionine, respectively) were selected and crossed with *trp1Δ* or *ade2Δ* cells. Tetrads resulting from a cross between *trp1Δ* and *ade2Δ* cells were dissected, yielding auxotrophic colonies for tryptophan and adenine. The obtained strains were crossed, and the resulting diploids were sporulated. After tetrad dissection, we obtained YMT43 and YMT44 colonies that were auxotrophic for both tryptophan and adenine (YMT43: *MAT* **a** *ura3Δ0 leu2Δ0 his3Δ1 met15Δ0 lys2Δ0 trp1Δ::KanMX ade2Δ::KanMX*; YMT44: *MAT*α *ura3Δ0 leu2Δ0 his3Δ1 met15Δ0 lys2Δ0 trp1Δ::KanMX ade2Δ::KanMX*).

YMT44 cells were crossed with *thr1Δ* or *arg1Δ* cells and the resulting diploids were sporulated. Spores with the following genotypes were selected: *MAT* **a** *ura3Δ0 leu2Δ0 his3Δ1 met15Δ0 lys2Δ0 trp1Δ::KanMX ade2Δ::KanMX thr1Δ::KanMX* (from a cross between YMT44 and *thr1Δ*) and *MAT*α *ura3Δ0 leu2Δ0 his3Δ1 met15Δ0 lys2Δ0 trp1Δ::KanMX ade2Δ::KanMX arg1Δ::KanMX* (from a cross between YMT44 and *arg1Δ*). These cells were crossed, and the resulting diploids were sporulated. After tetrad dissection, we obtained an auxotrophic strain for both threonine and arginine (*MAT*α *ura3Δ0 leu2Δ0 his3Δ1 met15Δ0 lys2Δ0 trp1Δ::KanMX ade2Δ::KanMX thr1Δ::KanMX arg1Δ::KanMX*). This strain was crossed with *tyr1Δ* cells, and the resulting diploids were sporulated. Spores auxotrophic for lysine, tryptophan, adenine, threonine, arginine, and tyrosine were identified, yielding YMT53 (*MAT*α) and YMT54 (*MAT* **a**). The genotypes of YMT53 and YMT54 were as follows: YMT53: *MAT*α *ura3Δ0 leu2Δ0 his3Δ1 met15Δ0 lys2Δ0 trp1Δ::KanMX ade2Δ::KanMX thr1Δ::KanMX arg1Δ::KanMX tyr1Δ::KanMX*; YMT54: *MAT* **a** *ura3Δ0 leu2Δ0 his3Δ1 met15Δ0 lys2Δ0 trp1Δ::KanMX ade2Δ::KanMX thr1Δ::KanMX arg1Δ::KanMX tyr1Δ::KanMX*. YMT55 was generated by crossing YMT53 and YMT54.

**Fig. 2:**
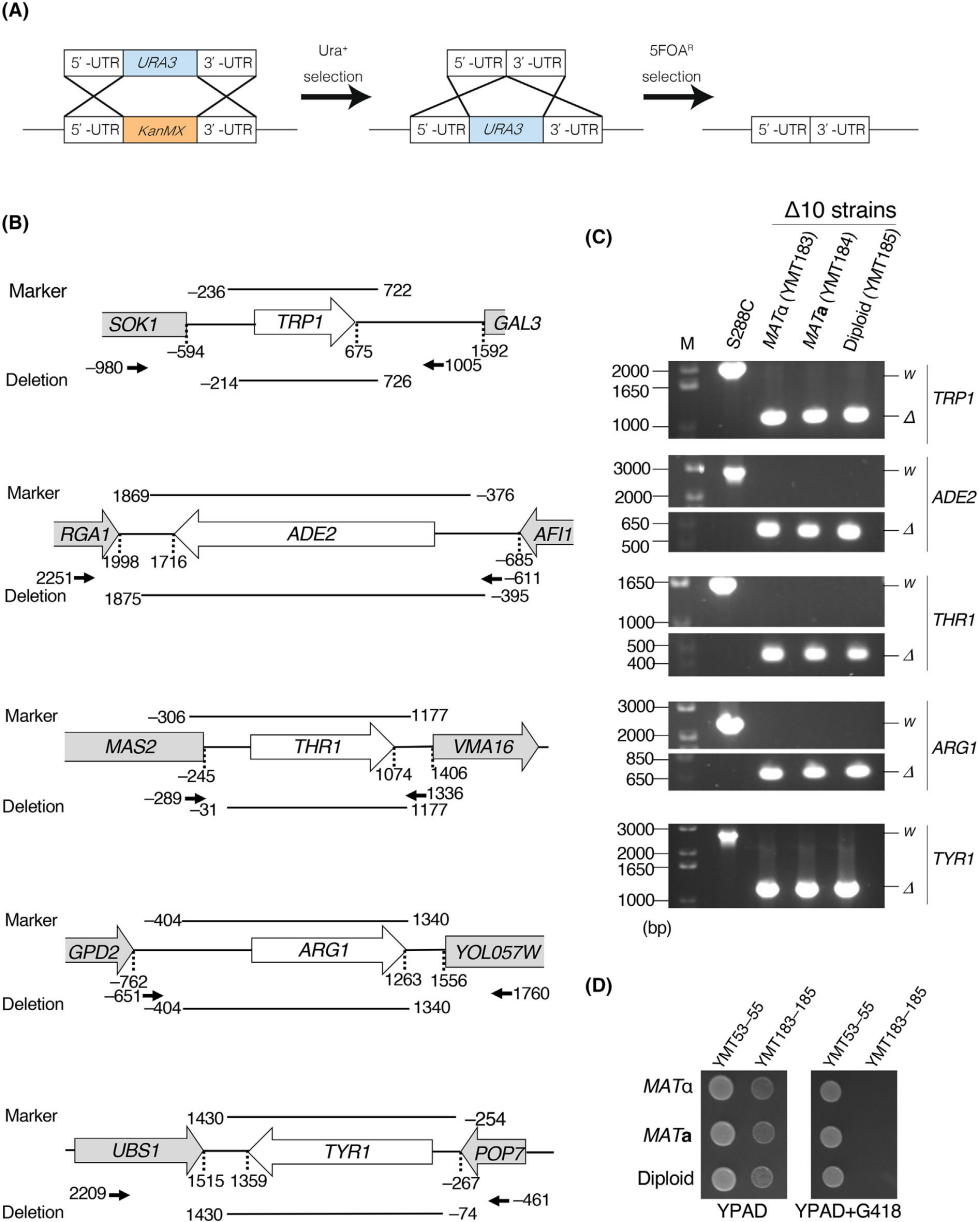
Removal of antibiotic markers. (A) Method of removing the antibiotic marker from a target locus. A DNA fragment of the *URA3* ORF flanked by upstream and downstream regions of the target auxotrophic marker gene was transformed into Δ10 cells, and cells were grown on media lacking uracil. The resulting Ura + cells were selected. Ura + cells were further transformed by a DNA fragment that contained upstream and downstream regions of the target auxotrophic marker gene, then grown on SD + 10 plates containing 5‐FOA. The resulting colonies on media containing 5‐FOA (FOA^R^ cells) were selected; the cells were expected to have lost the antibiotic marker from the target locus. (B) Deleted regions in each auxotrophic marker and overlap with vector segments. The genomic region encompassing each auxotrophic marker gene is presented. The definitions of nucleotide numbers were based on assigning +1 to the A of the first ATG in the auxotrophic marker ORF. Lines above each marker locus indicate genomic regions that are cloned into the corresponding pESC vector (Fig. 3B). Lines under each marker locus indicate deleted regions. Arrows indicate primer sets used in PCR to confirm deletions in each locus (C), and the nucleotide numbers corresponding to the 5′‐nucleotide of each primer are shown. (C) Removal of the antibiotic marker was confirmed by PCR. M: 10‐kbp DNA marker; *W*: wild‐type allele; *Δ*: deletion allele. (D) The new Δ10 strains, YMT183–YMT185, were sensitive to G418. Cells were spotted and cultured on YPAD or YPAD + G418 plates for 1 day at 27 °C.

A schematic diagram of *KanMX* removal from auxotrophic marker gene loci in YMT53 and YMT54 is shown in Fig. 2A (see also Fig. S1).

To remove *KanMX* from the *ADE2* locus, regions upstream and downstream of the *ADE2* open reading frame (ORF) were PCR‐amplified using the M29/M413 and M30/M414 primer sets, respectively. The resultant PCR DNA fragments and primer set M29/M30 were used for the second PCR, resulting in DNA fragments containing *URA3* flanked by regions upstream and downstream of the *ADE2* ORF. These DNA fragments were transformed into yeast, and cells were grown on SD + 10 − Ura plates, resulting in cells carrying the *ade2Δ::URA3* allele. To remove the integrated *URA3* from the *ADE2* locus, the regions upstream and downstream of the *ADE2* ORF were PCR‐amplified using the M29/M441 and M30/M440 primer sets, respectively. A second PCR was performed using the upstream and downstream *ADE2* regions as templates and the primer set M29/M30, resulting in DNA fragments containing the upstream and downstream *ADE2* flanking regions. The obtained DNA fragments were transformed into yeast, and the cells were grown on SD + 10 media containing 5‐fluoroorotic acid (5‐FOA), resulting in cells carrying the *ade2Δ4* allele.

Removal of *KanMX* from *TYR1*, *TRP1*, *ARG1*, and *THR1* loci was performed in a similar manner. For the construction of DNA fragments containing the *URA3* ORF, PCRs were performed using the following primer sets: *TYR1*, M36/M437 and M402/M438; *TRP1*, M333/M422 and M334/M423; *ARG1*, M40/M395 and M396/M411; and *THR1*, M38/M393 and M39/M390. The second PCRs were performed using the PCR‐amplified DNA fragments and the following primer sets: *TYR1*, M36/M402; *TRP1*, M333/M334; *ARG1*, M40/M411; and *THR1*, M38/M39. For the construction of DNA fragments used to remove *URA3* from the target locus, PCRs were performed using the following primer sets: *TYR1*, M36/M470 and M37/M469; *TRP1*, M433/M435 and M334/M434; *ARG1*, M40/M468 and M401/M467; and *THR1*, M38/M487 and M39/M486. Then, second PCRs corresponding to each of these were performed using the respective PCR‐amplified DNA fragments and the following primer sets: *TYR1*, M36/M37; *TRP1*, M334/M433; *ARG1*, M40/M401; and *THR1*, M38/M39.

The resulting YMT183 (*MAT*α) and YMT184 (*MAT* **a**) strains were crossed to produce the diploid strain, YMT185.

### Restoration of *ADE2* , *TRP1* , *TYR1* , *ARG1* , and *THR1* in Δ10 strains

DNA fragments containing *ADE2*, *TRP1*, *TYR1*, *ARG1*, and *THR1* were amplified via PCR using the yeast genome as a template along with primer sets T289/T290, M334/M433, M42/M402, M401/T291, and T292/T293, respectively. Δ10 cells (YMT183 and YMT184) were transformed with these DNA fragments, and cells were grown on media lacking the corresponding nutrients (e.g., cells transformed with *ADE2* fragments were grown on an SD + 10 − Ade plate). Ade^+^, Trp^+^, Tyr^+^, Arg^+^, and Thr^+^ colonies were picked up and restoration of these genes was confirmed by PCR (detailed in the PCR analysis section below).

### Doubling time calculation

Cells grown overnight in YPAD were diluted to OD_600_ = 0.1 in 3 mL of YPAD and then cultured at 30 °C. OD_600_ was measured every 2 h. Doubling time was measured from the exponential growth phase using graphpad prism version 10.2.3 (Dotmatics, Boston, MA, USA).

### Construction of pESC plasmids with new markers

For the construction of pESC‐MET^Pro^, pESC‐ADE^Pro^, and pESC‐THR^Pro^, PCRs were performed using the yeast genome as template DNA and the M34/M35, M29/M33, and M38/M39 primer sets, respectively. The amplified fragments were cloned into the PstI and StuI sites of pESC‐URA. To construct pESC‐LYS^Pro^, pESC‐TYR^Pro^, and pESC‐ARG^Pro^, PCRs were performed using the yeast genome as template DNA and the M31/M32, M36/M37, and M40/M41 primer sets, respectively. The amplified fragments were cloned into NdeI and StuI sites of pESC‐URA.

To construct pESC‐THR, the NheI site present in the *THR1* marker on pESC‐THR^Pro^ was removed by site‐directed mutagenesis using the M425/M426 primer sets. The remaining *URA3* ORF and promoter sequences were removed by inverse PCR using M446/M448 and M38/442 primer sets, respectively.

To construct pESC‐ADE, the BglII site present in the *ADE2* marker on pESC‐ADE^Pro^ was removed by site‐directed mutagenesis using the M405/M406 primer set. The remaining *URA3* ORF and promoter sequences were removed by inverse PCR using M448/M460 and M442/455 primer sets, respectively.

To construct pESC‐ARG, the KpnI and ClaI sites present in the *ARG1* marker on pESC‐ARG^Pro^ were removed by site‐directed mutagenesis using the M451/M452 and M456/M457 primer sets, respectively. The remaining *URA3* ORF and promoter sequences were removed by inverse PCR using M41/M448 and M442/461 primer sets, respectively.

To construct pESC‐MET, the EcoRI site present on the *MET15* marker on pESC‐MET^Pro^ was removed by site‐directed mutagenesis using the M418/M419 primer sets. The remaining *URA3* ORF and promoter sequences were removed by inverse PCR using M448/M464 and M442/462 primer sets, respectively.

To construct pESC‐LYS, the BamHI, BglII, and XhoI sites present in the *LYS2* marker on pESC‐LYS^Pro^ were removed by site‐directed mutagenesis using the M407/M408, M416/M417, and M429/M430 primer sets, respectively. The remaining *URA3* ORF and promoter sequences were removed by inverse PCR using M32/M448 and M442/M443 primer sets, respectively.

To construct pESC‐TYR, the EcoRI, HindIII, SacI, and SpeI sites present in the *TYR1* marker on pESC‐TYR^Pro^ were removed by site‐directed mutagenesis using the M431/M432, M444/M445, M453/M454, and M458/M459 primer sets, respectively. The remaining *URA3* ORF and promoter sequences were removed by inverse PCR using M448/M473 and M442/M474 primer sets, respectively.

To replace the Myc sequence on the original plasmids with the HA sequence, the original plasmids were PCR‐amplified using the T181/T182 primer set to form a linear DNA product. A DNA fragment containing the HA sequence (T270) was inserted into the original Myc site using an InFusion HD cloning kit (Takara Bio, Inc., Tokyo, Japan). Replacement of the FLAG sequence with the V5 sequence was performed in a similar manner using the T190/T263 primer set and a DNA fragment containing the V5 sequence (T269).

### PCR analysis

To confirm the deletion of alleles of the auxotrophic marker genes in Δ10 strains (YMT53–YMT55), PCRs were performed using the genome of each strain as a template, along with primer sets flanking the target gene. The following primer sets were used: *URA3*, M335/M336; *LEU2*, M330/M331; *HIS3*, M328/M329; *MET15*, M34/M332; *TRP1*, M333/M334; *ADE2*, M327/M356; *LYS2*, M31/M32; *ARG1*, M40/M41; *THR1*, M38/M39; and *TYR1*, M36/M37.

To confirm the presence of the *trp1Δ4*, *ade2Δ4*, *thr1Δ2*, *arg1Δ0*, and *tyr1Δ0* alleles, PCRs were performed in a similar manner. The following primer sets were used: *TRP1*, M334/M433; *ADE2*, M29/M33; *THR1*, M409/M410; *ARG1*, M40/M401; and *TYR1*, M42/M402.

To confirm that Δ10 and Δ9 cells could maintain 10 and 9 types of pESC plasmids, respectively, PCRs were performed using DNA extracted from Δ10 and Δ9 cells transformed by these plasmids as templates, along with primer sets specific for marker segments on the plasmids. The primer sets were as follows: *URA3*, M354/M490; *LEU2*, M490/M525; *HIS3*, M350/M490; *TRP1*, M352/M490; *ADE2*, M460/M490; *MET15*, M419/M490; *LYS2*, M32/M490; *ARG1*, M452/M490; *THR1*, M426/M490; and *TYR1*, M431/M490.

To investigate whether interplasmid recombination occurred *in vivo*, PCRs were performed using DNA extracted from cells harboring 10 plasmids (YMT216, YMT218, YMT219, and YMT220) along with primer sets that could amplify DNA fragments encompassing two multiple cloning sites. The primer sets were as follows: *URA3*, T221/T316; *LEU2*, T221/T310; *HIS3*, T221/T309; *TRP1*, T221/T314; *ADE2*, T221/T307; *MET15*, T221/T312; *LYS2*, T221/T311; *ARG1*, T221/T308; *THR1*, T221/T313; and *TYR1*, T221/T315. The control template sets (empty plasmid/plasmids encoding luciferase constructs) were as follows: *URA3*, pESC‐URA/pMT440; *LEU2*, pMT455/pMT459; *HIS3*, pESC‐HIS/pMT460; *TRP1*, pMT457/pMT461; *ADE2*, pMT302/pMT462; *MET15*, pMT447/pMT463; *LYS2*, pMT305/pMT464; *TYR1*, pMT451/pMT465; *ARG1*, pMT303/pMT466; *THR1*, pMT449/pMT468.

### Western blotting

Growing cells in selective SD or SG media were harvested by centrifugation at 9000 ***g*** for 1 min, resuspended in 0.1 N NaOH, and incubated for 5 min at room temperature. The cells were harvested again at 9000 ***g*** for 1 min, resuspended, and boiled in SDS sample buffer. SDS/PAGE and western blotting were performed as described elsewhere [23, 24]. The antibodies used in this study are listed in Table S5.

## Results

### Construction of Δ10 strains

Using the Kyoto Encyclopedia of Genes and Genomes and Saccharomyces Genome Database (SGD), we searched for auxotrophic markers that were not used in the W303 and BY4741/BY4742 strains [25, 26]. We identified the candidate genes *THR1*, *ARG1*, and *TYR1* that encode enzymes essential for the biosynthesis of l‐threonine, l‐arginine, and l‐tyrosine, respectively [27]. Indeed, mutations of these genes result in auxotrophy for corresponding amino acids [28, 29, 30].

Starting with BY4743 [20], we aimed to create strains carrying mutations in a total of 10 auxotrophic marker genes: *URA3*, *LEU2*, *HIS3*, *MET15*, *TRP1*, *ADE2*, *LYS2*, *THR1*, *ARG1*, and *TYR1*. Figure 1A shows a schematic representation of this strategy. First, BY4743 cells were sporulated under starvation conditions and the resulting tetrads were segregated. Since BY4743 carries heterozygous deletions in *MET15* and *LYS2*, a strain carrying homozygous deletions in these two genes was obtained by selecting segregated cells that did not grow in either lysine‐ or methionine‐deficient media. This strain was further crossed with two other strains, one with *TRP1* deletion and the other with *ADE2* deletion, and the resulting diploid cells were sporulated and segregated. *URA3*, *LEU2*, *HIS3*, *MET15*, *LYS2*, and *TRP1* deletion strains, and *URA3*, *LEU2*, *HIS3*, *MET15*, *LYS2*, and *ADE2* deletion strains were obtained by auxotrophic selection. By crossing these two strains, strains carrying *URA3*, *LEU2*, *HIS3*, *MET15*, *LYS2*, *TRP1*, and *ADE2* deletions (YMT43/YMT44) were obtained.

Next, *THR1* and *ARG1* deletion alleles were introduced into YMT44 by crossing YMT44 with *thr1Δ* and *arg1Δ cells*, respectively. These cells were crossed with each other, and the resulting diploids segregated, yielding a strain carrying both *THR1* and *ARG1* deletions. This strain was crossed with the *TYR1* deletion strain and segregated. Among the segregated cells, we obtained Δ10 strains (YMT53/YMT54) strains that exhibited auxotrophic behavior for uracil, leucine, histidine, methionine, lysine, tryptophan, adenine, threonine, arginine, and tyrosine (Fig. 1A,B). The diploid Δ10 strain (YMT55) was obtained by crossing YMT53 and YMT54. Deletions in auxotrophic markers were confirmed using PCR (Fig. 1C).

Since *TRP1*, *ADE2*, *THR1*, *ARG1*, and *TYR1* deletion alleles contained the antibiotic marker cassette *KanMX*, which confers resistance to geneticin (G418), we removed the antibiotic marker from each locus using *URA3*/5‐FOA counterselection [31]. To exchange *KanMX* with *URA3*, Δ10 cells were transformed by a DNA fragment containing *URA3* flanked by 5′‐ and 3′‐untranslated regions (UTRs) of the target gene. Ura + colonies were selected on uracil‐deficient media. To remove *URA3* from the target locus, Ura + cells were further transformed by a DNA fragment containing 5′‐ and 3′‐UTRs of the target gene. Ura − colonies were selected on media containing 5‐FOA (Fig. 2A). In the latter transformation, several hundred base pairs (bp) of 5′‐ and 3′‐UTRs were removed from the target locus so that there was no homology between the marker segment in the commonly used plasmid and the deletion in the chromosome (Fig. 2B). By repeating this process, we removed the antibiotic markers from the *TRP1*, *ADE2*, *THR1*, *ARG1*, and *TYR1* loci to obtain YMT183 and YMT184 (Fig. 2C). The modified deletion alleles of *trp1*, *ade2*, *thr1*, *arg1*, and *tyr1* were designated *trp1Δ4*, *ade2Δ4*, *thr1Δ2*, *arg1Δ0*, and *tyr1Δ0*, respectively. The diploid strain YMT185 was obtained by crossing YMT183 and YMT184. We confirmed that YMT183–YMT185 no longer exhibited G418 resistance (Fig. 2D).

### Construction of pESC plasmids with new markers

**Fig. 3:**
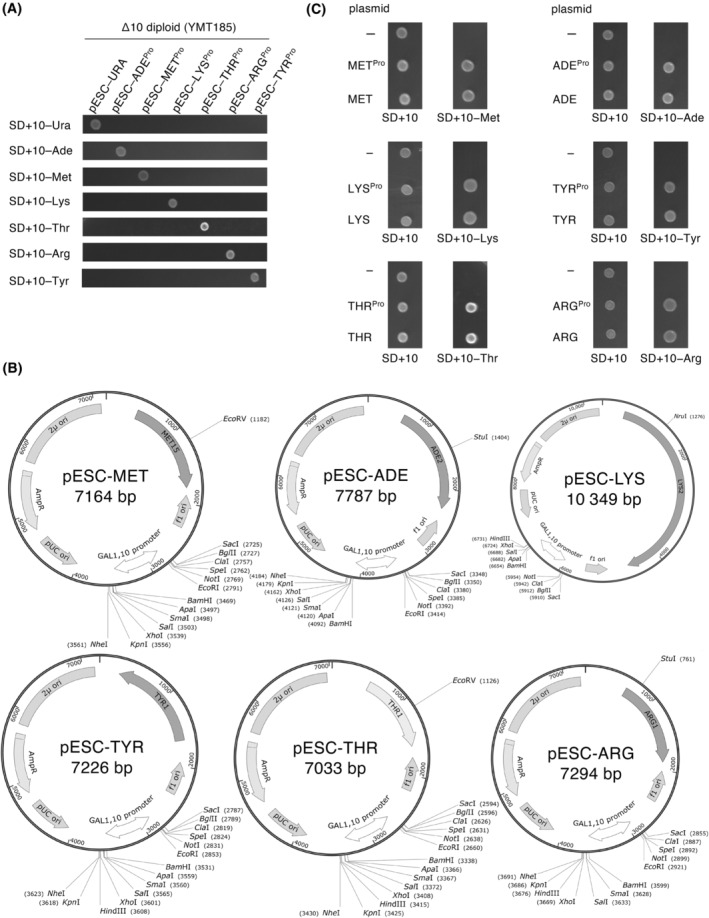
Construction of pESC plasmids with new marker genes. (A) Cells carrying a pESC plasmid do not exhibit auxotrophy for the nutrient corresponding to the auxotrophic marker gene on the plasmid. Cells were spotted and cultured on the indicated plates for 2 days at 27 °C. (B) Maps of new pESC plasmids. Unique restriction sites are shown. (C) Confirmation that modified pESC plasmids can be used for auxotrophic selection. Cells were spotted and cultured on the indicated plates for 2 days at 27 °C.

pESC plasmids are yeast 2 micron expression vectors containing two multiple cloning sites that enable the co‐expression of two genes [32]. Commercially available pESC‐URA, pESC‐LEU, pESC‐HIS, and pESC‐TRP plasmids contain *URA3*, *LEU2*, *HIS3*, and *TRP1* as selectable markers. We constructed new pESC plasmids for use with Δ10 strains by replacing the marker segment of pESC‐URA, as follows: pESC‐MET^Pro^ (*MET15* marker), pESC‐ADE^Pro^ (*ADE2* marker), pESC‐THR^Pro^ (*THR1* marker), pESC‐LYS^Pro^ (*LYS2* marker), pESC‐TYR^Pro^ (*TYR1* marker), and pESC‐ARG^Pro^ (*ARG1* marker) (‘Pro’ denotes a prototype, because these plasmids were modified as described below). Transformation of Δ10 cell lines using these plasmids yielded colonies on the corresponding selective media (Fig. 3A).

To increase the number of unique restriction sites in the multiple cloning sites, site‐directed silent mutations were introduced into the auxotrophic markers. Specifically, the following were destroyed: the NheI site (+1054) of *THR1*; BglII site (+592) of *ADE2*; KpnI (+269) and ClaI (+806) sites of *ARG1*; EcoRI site (+415) of *MET15*; BglII (+387), XhoI (+2864), and BamHI sites (+3246) of *LYS2*; and HindIII (−105), SpeI (+429), EcoRI (+963), and SacI sites (+1192) of *TYR1*. The following modified pESC plasmids were obtained: pESC‐MET, pESC‐ADE, pESC‐LYS, pESC‐TYR, pESC‐THR, and pESC‐ARG (Fig. 3B). Transformants with these modified pESC plasmids were grown on the corresponding selective media, similar to the prototype pESC plasmids (Fig. 3C). We also constructed plasmids in which the FLAG and Myc sequences were replaced with V5 and HA, respectively (pMT446–pMT451 and pMT455–pMT458, Table S2).

### Establishment of a novel plasmid‐based yeast expression system that enables co‐expression of more than 10 different genes

**Fig. 4:**
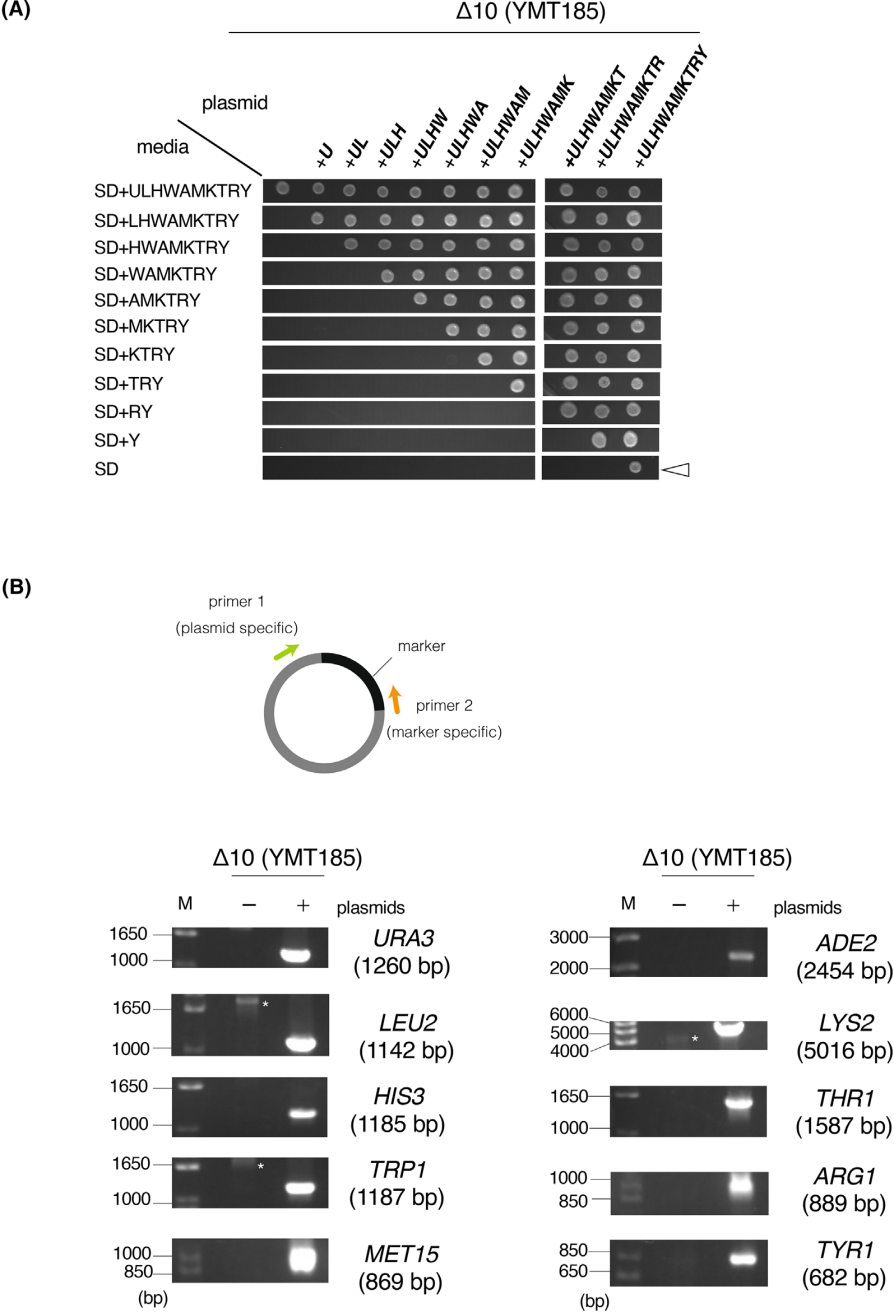
Confirmation that Δ10 cells can carry 10 pESC plasmids. (A) Sequential introduction of pESC plasmids into Δ10 cells. Δ10 cells (YMT185) were serially transformed by pESC plasmids. Transformed cells were spotted and cultured on the indicated plates for 2 days at 27 °C. pESC plasmids are indicated as follows: *U* = pESC‐URA, *L* = pESC‐LEU, *H* = pESC‐HIS, *W* = pESC‐TRP, *A* = pESC‐ADE, *M* = pESC‐MET, *K* = pESC‐LYS, *T* = pESC‐THR, *R* = pESC‐ARG, *Y* = pESC‐TYR. The arrowheads represent cells harboring 10 pESC plasmids. (B) Confirmation that cells grown on SD harbored all 10 pESC plasmids. PCRs were performed using DNA extracted from YMT185 or cells that grow on SD as a template along with primer sets specific for the marker segment on the plasmid. Expected sizes of PCR products are indicated in each electrogram. Asterisks indicate non‐specific bands. M, 10‐kbp DNA marker.

We next examined whether Δ10 cells could carry all 10 plasmids. The plasmids were used to sequentially transform Δ10 cells (YMT185) and successfully obtain cells that grew on media lacking the corresponding amino acids, adenine, and uracil (Fig. 4A, SD). PCRs were performed using DNA extracted from cells grown on SD as a template along with primer sets specific for the marker segment on each plasmid. PCR products of the expected sizes were amplified, demonstrating that the cells grown on SD carried 10 plasmids (Fig. 4B).

**Fig. 5:**
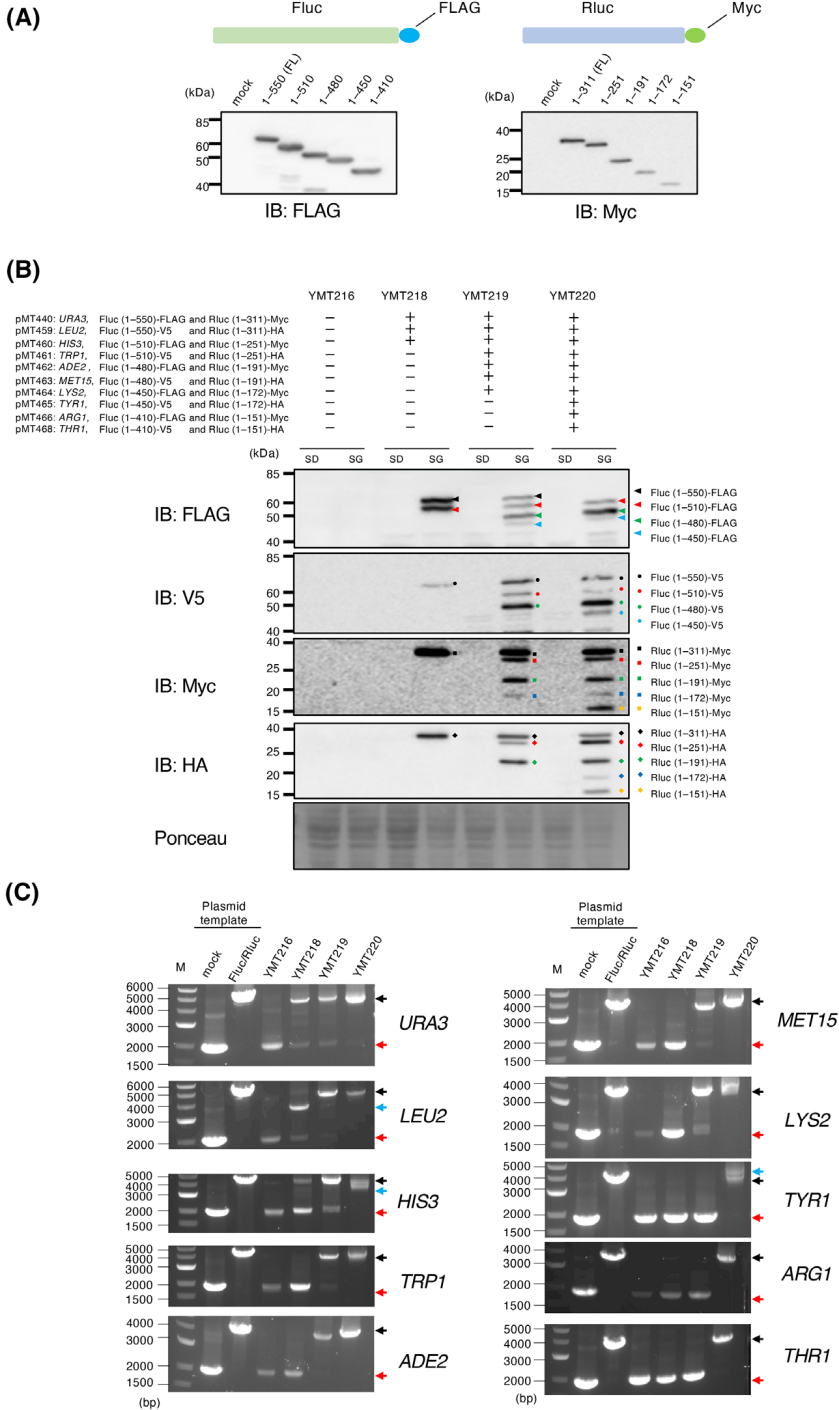
Validation of Δ10‐based expression system. (A) Δ10 strain (YMT185) transformed with plasmids encoding indicated luciferase constructs were grown in galactose‐containing media. Whole‐cell extracts were analyzed using the indicated antibodies to detect constructs. For detection of firefly luciferase constructs, cells (YMT185) transformed with pMT440 (full length), pMT454 (aa 1–510), pMT444 (aa 1–480), pMT453 (aa 1–450), and pMT445 (aa 1–410) were analyzed. Detection of Renilla luciferase was performed in similar manner, using cells transformed with pMT440 (full length), pMT444 (aa 1–251), pMT445 (aa 1–191), pMT441 (aa 1–172), and pMT442 (aa 1–151). FL, full length. (B) YMT216, YMT218, YMT219, and YMT220 were grown in glucose or galactose‐containing media. Whole‐cell extracts were analyzed by immunoblotting as in (A). (C) Confirmation that cells harbored the expected set of plasmids. PCRs were performed using empty plasmid (mock), plasmids encoding luciferase constructs (Fluc/Rluc), and DNA extracted from indicated strains as a template along with primer sets that can amplify DNA fragments encompassing two multiple cloning sites. Black arrows indicate the DNA fragments amplified from intact plasmids encoding both firefly and Renilla luciferase constructs. Red arrows indicate the DNA fragments amplified from empty plasmids. Blue arrows indicate the DNA fragments that were not assigned to both empty and intact plasmids. The control template sets (empty plasmid/plasmids encoding luciferase constructs) were as follows: *URA3*, pESC‐URA/pMT440; *LEU2*, pMT455/pMT459; *HIS3*, pESC‐HIS/pMT460; *TRP1*, pMT457/pMT461; *ADE2*, pMT302/pMT462; *MET15*, pMT447/pMT463; *LYS2*, pMT305/pMT464; *TYR1*, pMT451/pMT465; *ARG1*, pMT303/pMT466; *THR1*, pMT449/pMT468. M, 10‐kbp DNA marker.

Because each plasmid enables the co‐expression of two genes, this strain has the potential to co‐express a maximum of 20 different genes. To confirm this, we first queried truncated constructs of firefly luciferase (Fluc) and Renilla luciferase (Rluc), whose expression was detected in Δ10 cells (Fig. 5A). Next, we introduced plasmids encoding these constructs into Δ10 cells. The resulting three strains, YMT218, YMT219, and YMT220, were expected to co‐express 6, 14, and 20 model constructs, respectively (Table S1). To test this hypothesis, we investigated the expression of each construct after galactose induction. Western blot analysis showed that most of the constructs were expressed: 5, 14, and 17 bands were observed in YMT218, YMT219, and YMT220, respectively. While we detected the expected number of 14 protein bands from lysates of YMT219, the expression of several constructs was not detected in YMT218 and YMT220: Myc‐tagged Rluc (aa 1–251) in YMT218 and FLAG‐tagged Fluc (full length), FLAG‐tagged Fluc (aa 1–410), and V5‐tagged Fluc (aa 1–410) in YMT220 (Fig. 5B). This could hypothetically be due to defects in plasmids used. For Myc‐tagged Rluc (aa 1–251) and FLAG‐tagged Fluc (full length), which were not detected in YMT218 and YMT220, respectively, these constructs were detected in other strains (e.g., full‐length Fluc was detected in YMT218 and YMT219). For FLAG‐tagged Fluc (aa 1–410) and V5‐tagged Fluc (aa 1–410), which were not detected in YMT220, we transformed Δ10 (YMT185) with pMT466 and pMT468. Western blot analysis detected Fluc (aa 1–410) in both transformants (Fig. S2). Taken together, the possibility of defects in plasmids used in Fig. 5B can be ruled out.

Because the sequence of plasmids constructed in this study was identical to each other except for marker segments, there is a substantial risk of interplasmid recombination, which leads to loss of luciferase segments. To investigate this, we performed PCR analysis using primer sets that can amplify DNA fragments encompassing two multiple cloning sites. The results indicate that interplasmid recombination‐mediated gene loss can indeed occur; in addition to PCR‐amplified fragments from intact plasmids encoding luciferase constructs, fragments whose sizes were equivalent to those from empty plasmids were observed (e.g., PCR using DNA from YMT218 as a template with primer sets for amplifying gene segments on pESC‐URA, Fig. 5C). We also detected DNA fragments that were not assigned to either empty or intact plasmids (*LEU2*, *HIS3*, and *TYR1*, Fig. 5C). We speculate that these unassigned DNA fragments were amplified from plasmids produced by interplasmid recombination. For Myc‐tagged Rluc (aa 1–251) in YMT218, which was not detected in western blot analysis, the PCR‐amplified fragment whose size corresponded to that of intact plasmid encoding Myc‐tagged Rluc (aa 1–251) was detected (Fig. 5C, *HIS3*). Likewise, for FLAG‐tagged Fluc (full length), FLAG‐tagged Fluc (aa 1–410), and V5‐tagged Fluc (aa 1–410) in YMT220, PCR fragments that corresponded to intact plasmids were detected (Fig. 5C, *URA3*, *ARG1*, and *THR1*). At present, we have not yet elucidated the mechanism underlying variation in the expression levels.

### Re‐engineering of Δ10

**Fig. 6:**
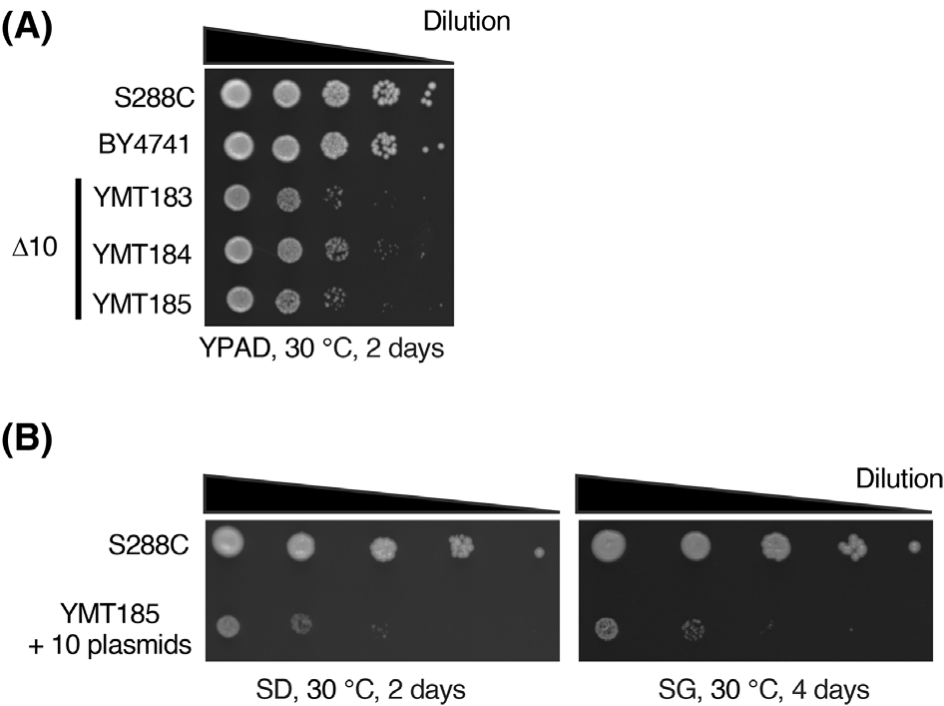
Growth of Δ10. (A) Cells were spotted and cultured on YPAD plates for 2 days at 30 °C. (B) Growth of S288C and Δ10 strain (YMT185) harboring 10 plasmids (Fig. 4) on SD or SG was analyzed as in (A).

**Fig. 7:**
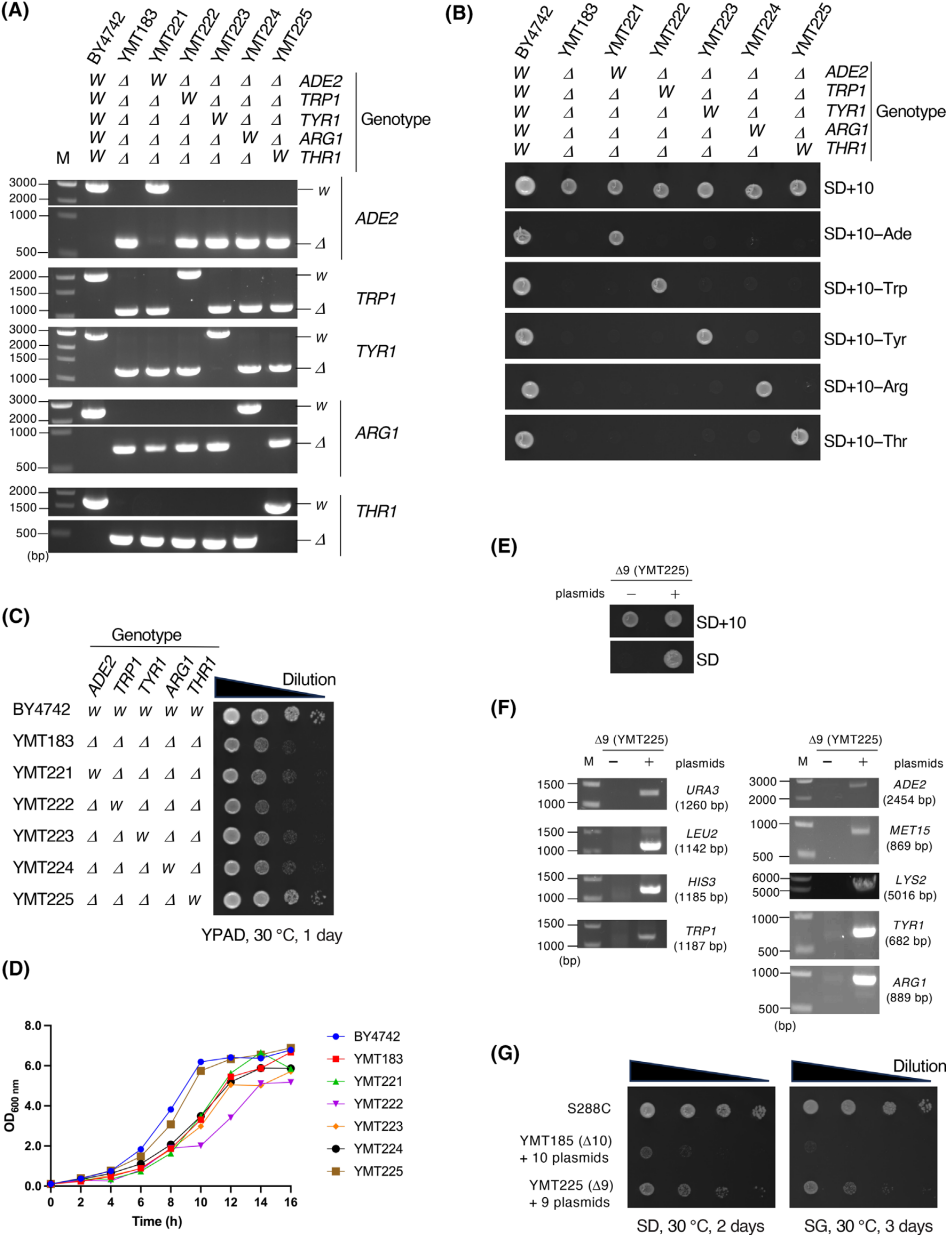
Re‐engineering of Δ10. (A) Restoration of *ADE2*, *TRP1*, *TYR1*, *ARG1*, and *THR1* in YMT183 was confirmed by PCR as in Fig. 2C. M, 10‐kbp DNA marker; *W*, wild‐type allele; *Δ*, deletion allele. (B) Cells were spotted and cultured on the indicated plates for 2 days at 30 °C. *W*, wild‐type allele; *Δ*, deletion allele. (C) Cells were spotted and cultured on YPAD plates for 1 day at 30 °C. *W*, wild‐type allele; *Δ*, deletion allele. (D) Growth curves of indicated strains. Cells were cultured in liquid YPAD media and OD_600_ was measured every 2 h. (E) YMT225 was transformed with nine plasmids: pESC‐URA, pESC‐LEU, pESC‐HIS, pESC‐TRP, pESC‐ADE (pMT302), pESC‐MET (pMT304), pESC‐LYS (pMT305), pESC‐TYR (pMT315), and pESC‐ARG (pMT303). YMT225 (−) and YMT225 transformed with nine plasmids (+) were spotted and cultured on the indicated plates for 2 days at 30 °C. (F) Confirmation that cells harbored all nine pESC plasmids. PCRs were performed using DNA extracted from cells that grow on SD (E) as a template along with primer sets specific for the marker segment on the plasmid. Expected sizes of PCR products are indicated in each electrogram. M, 10‐kbp DNA marker. (G) Cells were spotted and cultured on the indicated plates for 2–3 days at 30 °C.

Finally, we investigated the fitness of Δ10 cells. Δ10 (YMT183–185) cells showed poor growth compared with BY4741 and S288C cells (Fig. 6A). We also analyzed the growth of Δ10 cells harboring these 10 plasmids. Again, cells harboring the 10 plasmids showed poor growth on both glucose‐ and galactose‐containing media as compared to the S288C cells (Fig. 6B). To investigate which gene deletion(s) is responsible for the growth defect, we restored deleted genes (i.e., *ADE2*, *TRP1*, *THR1*, *ARG1*, and *TYR1*) in YMT183 (*MAT*α) and analyzed the growth (Fig. 7A,B). The result showed that the growth of *THR1*‐restored strain (YMT225) was roughly comparable to that of BY4742, indicating that the growth defect is primarily attributed to *thr1Δ2* (Fig. 7C,D, and Table 1). We also restored *THR1* in YMT184 (*MAT* **a**), yielding YMT226, and again observed an improvement in growth (Fig. S3A,B). *THR1*‐restored Δ10 strains were designated as Δ9 strains. Δ9 strains are able to carry 9 plasmids (Fig. 7E,F). The growth of Δ9 cells harboring 9 plasmids was greatly improved compared to the Δ10 counterpart, though not to the same extent as in S288C (Fig. 7G).

**Table 1:** Doubling time of re‐engineered strains (related to Fig. 7D).

Strain	Doubling time (h)^a^
BY4742	2.0 ± 0.02
YMT183	2.3 ± 0.12
YMT221	2.4 ± 0.08
YMT222	2.6 ± 0.11
YMT223	2.4 ± 0.06
YMT224	2.4 ± 0.10
YMT225	2.1 ± 0.09

^a^ Values are the average of three independent culture ± standard error of the mean.

## Discussion

In this study, we established a novel expression system in *S. cerevisiae* that enables the co‐expression of more than 10 genes. The commonly used yeast strains BY4741 and BY4742 carry *ura3Δ0*, *leu2Δ0*, and *his3Δ1*; the former carries *met15Δ0* whereas the latter carries *lys2Δ0* [20]. Another widely used strain, W303, carries *leu2‐3112*, *trp1‐1*, *ura3‐1*, *ade2‐1*, and *his3‐11,15* auxotrophic markers [19].

In addition to these commonly used auxotrophic markers, we successfully introduced deletion of *TYR1*, *ARG1*, and *THR1*. To our knowledge, this is the first study that exploited these genes as selection markers, though one previous study has indicated the potential of *TYR1* as a selectable marker [33]. Owing to this limited number of markers, the maximum numbers of plasmids that BY4741/BY4742 and W303 can maintain by auxotrophic selection are four and five, respectively. Thus, the Δ10‐ and Δ9‐based expression system developed in this study greatly expands the number of plasmids that can be maintained in cells via auxotrophic selection.

In addition to being a suitable model organism in the field of basic biology, budding yeast is utilized as a cell factory for producing high‐value compounds, such as fuels, food ingredients, and biopharmaceuticals [12, 13, 14, 15, 16, 17, 18, 34]. One of the most successful examples of this mode of production is that of human insulin, which is an essential biopharmaceutical for the treatment of diabetes [35]. Glucagon, human serum albumin, and hepatitis B virus vaccines are among the other biopharmaceuticals produced by budding yeast, as reviewed by Walker & Pretorius and Huang *et al*. [8, 34]. Recently, the biopharmaceuticals produced by budding yeast have expanded beyond recombinant peptides and proteins. Galanie *et al*. established the biosynthesis of opioid compounds thebaine and hydrocodone in budding yeast. In their study, large DNA fragments encoding genes involved in opioid biosynthesis (more than 20 genes in total) were integrated into the yeast genome to reconstitute opioid biosynthesis pathways. However, the construction of such large DNA fragments and their subsequent integration into the yeast genome is technically difficult. In contrast, the multi‐expression system constructed in this study is relatively easy to use because it is based on the classic methodology of the introduction of plasmids into yeast cells by auxotrophic complementation. Although the sequential introduction of plasmids is a time‐consuming process, we believe that the novel multi‐expression system established here will be a useful platform for yeast cell factories.

Using the current expression system, we accomplished co‐expression of 17 genes (Fig. 5B, YMT220). Meanwhile, several luciferase constructs were not detected. PCR analysis is indicative of interplasmid recombination events, which may lead to loss of constructs (Fig. 5C). However, PCR‐amplified fragments whose sizes corresponded to those amplified from intact plasmids encoding luciferase constructs were also observed. At present, we have not elucidated the basis of failure to express some constructs. A possible reason for this is the loss of short DNA segments essential for expression (e.g., epitope tags) due to recombination, which is difficult to detect by primer sets used in Fig. 5C. Another possible reason is the difference in the copy number of each plasmid, as the selection marker used strongly affects plasmid copy number, which contributes to variance in expression levels. A previous study reported that there is correlation between growth and the additional base pairs in cells [36]. Based on this finding, the copy number of pMT464 (pESC‐LYS encoding luciferase constructs) is thought to be the lowest, as pESC‐LYS is the largest in size (Fig. 3B). However, both firefly and Renilla luciferase constructs encoded by pMT464 (pESC‐LYS backbone) were detected in YMT219 and YMT220, while luciferase constructs on pESC‐ARG and pESC‐THR, both of which are smaller in size compared to pESC‐LYS, were not detected (Figs 3B and 5B). We speculate that the former case (interplasmid recombination) is more likely to result in failure of gene expression. Meanwhile, it is important to reduce the plasmid burden to ensure cell fitness and heterologous gene expression. Centromeric plasmids, which maintain low copy numbers per cell (one or two copies per cell) [7, 37], are beneficial for both improving growth and regulating the expression levels of plasmid‐encoded genes. Plasmids containing different promoters are also a feasible strategy for improving variance in expression levels.

Finally, we investigated the growth of Δ10 cells. Our results showed that Δ10 strains exhibited poor growth compared to the parent strain BY4741 and the ancestral strain S288C (Fig. 6A). To identify which gene deletion(s) is responsible for the growth defect, we restored deleted genes and found that the poor growth is mainly attributed to *thr1Δ2* (Fig. 7C). Because the growth of *THR1* plasmid‐complemented Δ10 remained unchanged (Fig. 6B), we speculate that decreased cell fitness of Δ10 is mainly attributed to deletion in *THR1* genomic locus, rather than loss of Thr1 expression, the former of which may affect the transcription of neighboring genes. In the threonine pathway, *THR4*, which encodes threonine synthase, may be a candidate for an alternative auxotrophy marker, because *THR4* deletion causes auxotrophy for threonine but does not affect growth [38]. However, it should be noted that *THR4* mutation increases sensitivity to various stresses [39, 40]. For *ARG1* and *TYR1*, few studies investigated the effect of *ARG1* and *TYR1* deletion on cell fitness. Past genome‐wide studies have indicated that deletion of *TYR1* causes growth defect while *ARG1* deletion does not [41, 42]. Thus, restoration of *TYR1* may contribute to further improvement of growth. It should be noted that we analyzed the cell fitness of Δ10 and Δ9 solely by growth. A multifaceted approach is needed to extensively evaluate the use of Δ10‐ and Δ9‐based experimental system.

Because Δ9 strains (YMT225 and YMT226) are able to carry 9 plasmids, accompanied by improved growth compared to Δ10, we propose to use Δ9 strains unless the introduction of 10 plasmids is required.

## Conclusions

In summary, we have developed a novel plasmid‐based experimental system in yeast that enables the co‐expression of more than 10 genes. Although improvements are required, this method holds promise as a tool for building novel yeast cell factories.

## Conflict of interest

The authors declare no conflict of interest.

### Peer review

The peer review history for this article is available at https://www.webofscience.com/api/gateway/wos/peer‐review/10.1002/2211‐5463.13893.

## Author contributions

TM designed the project, performed most of the experiments, analyzed and interpreted the data, prepared the figures, and wrote the manuscript. GD and TN performed the experiments, analyzed and interpreted the data, and wrote the manuscript.

## Supporting information


**Fig. S1.** Schematic for construction of *ade2Δ4*, *tyr1Δ0*, *trp1Δ4*, *arg1Δ0*, and *thr1Δ2*.
**Fig. S2.** Confirmation of the expression of Fluc constructs from pMT466 and pMT468.
**Fig. S3.** Re‐engineering of YMT184.

**Table S1.** Strains used in this study.
**Table S2.** Plasmids constructed and used in this study.
**Table S3.** Primers used in this study.
**Table S4.** Media used in this study.
**Table S5.** Antibodies used in this study.

## Data Availability

The data that support the findings of this study are available from the corresponding author, TM (tmatsuzaki@med.nagoya-u.ac.jp or matsuzaki.tetsuo.x5@f.mail.nagoya-u.ac.jp), upon request.
