# Supplementary material for: A novel plasmid‐based experimental system in Saccharomyces cerevisiae that enables the introduction of 10 different plasmids into cells
Source: FEBS Open Bio. 2024 Oct 10;14(12):1955–71. doi: 10.1002/2211-5463.13893 (PMC11609594; doi:10.1002/2211-5463.13893)
Supplement: Supplementary file 1 — Fig. S1. Schematic for construction of ade2Δ4, tyr1Δ0, trp1Δ4, arg1Δ0, and thr1Δ2. Fig. S2. Confirmation of the expression of Fluc constructs from pMT466 and pMT468. Fig. S3. Re‐engineering of YMT184. [file FEB4-14-1955-s001.pdf]

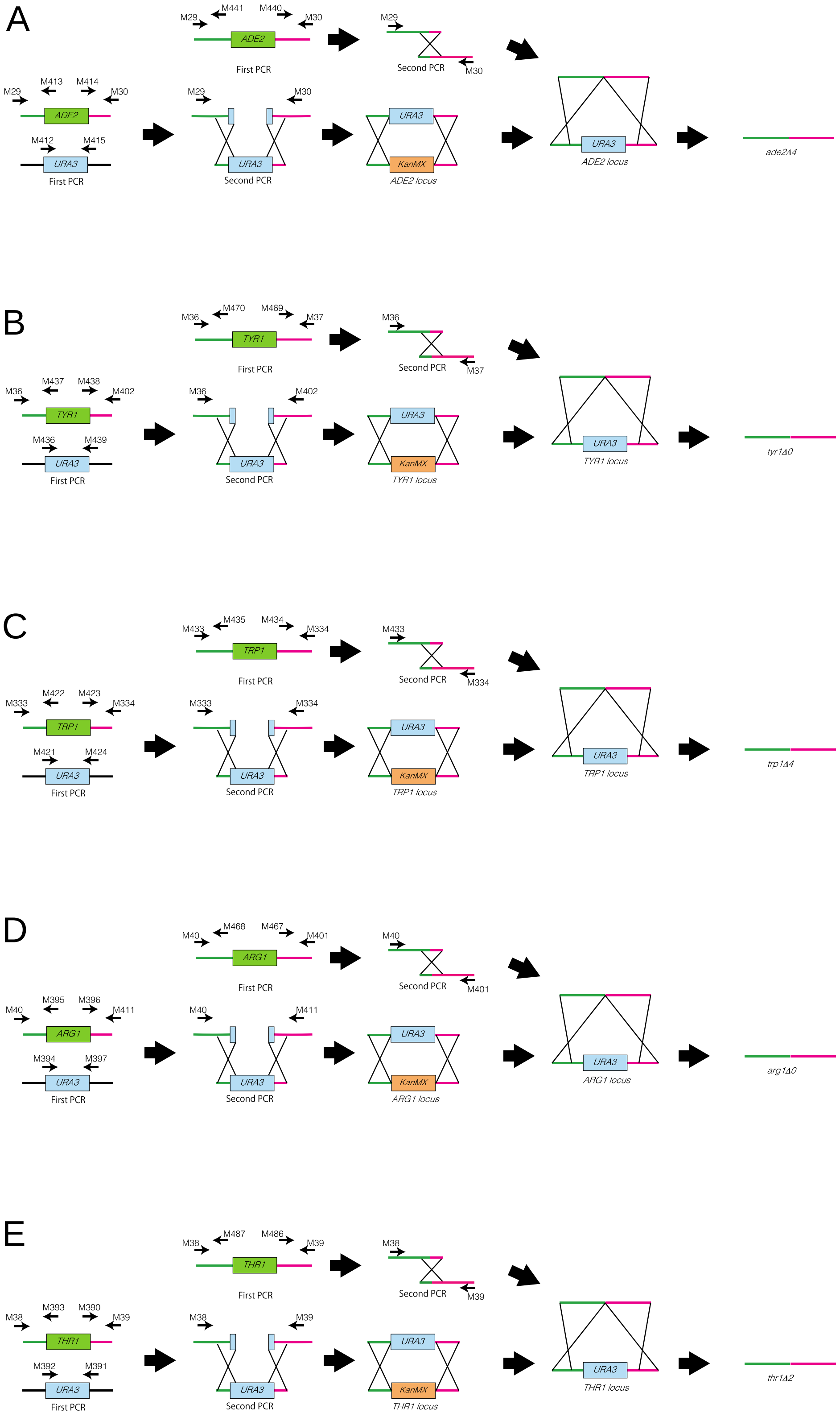

Fig. S1. Schematic for construction of *ade2Δ4*, *tyr1Δ0*, *trp1Δ4*, *arg1Δ0*, and *thr1Δ2*.

The methods for the construction of *ade2Δ4* (A), *tyr1Δ0* (B), *trp1Δ4* (C), *arg1Δ0* (D), and *thr1Δ2* (E) are described. Arrows indicate the primer (see also Table S3).

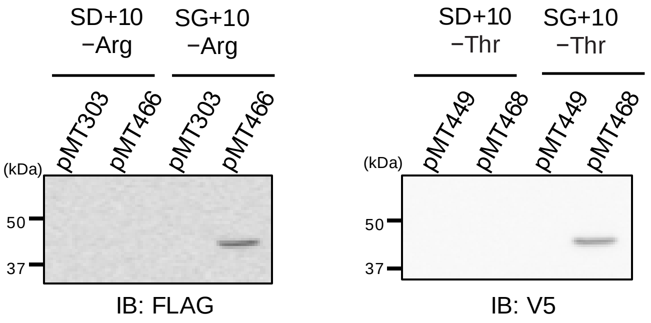

Fig. S2. Confirmation of the expression of Fluc constructs from pMT466 and pMT468.

Δ10 (YMT185) transformed with indicated plasmids were grown in SD and SG selective media.

Whole-cell extracts were analyzed by immunoblotting as in Fig. 5B.

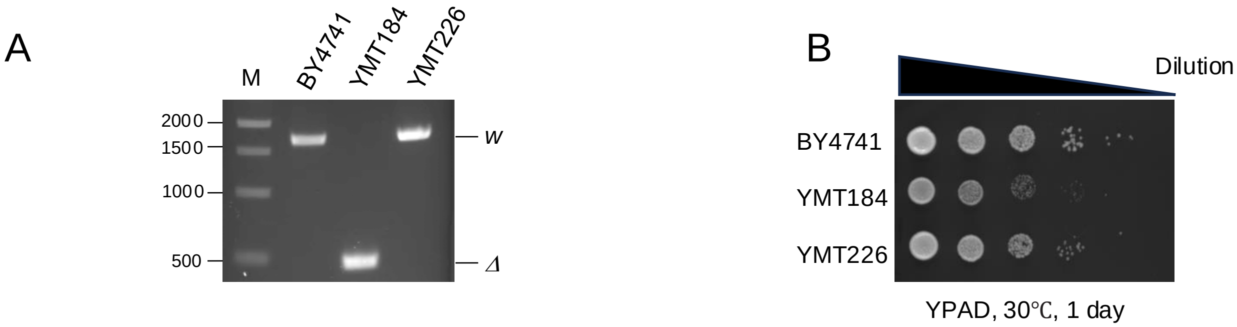

Fig. S3. Re-engineering of YMT184.

(A) Restoration of the *THR1* in YMT184 was confirmed by PCR as in Fig. 2C. M: 10-kbp DNA marker; *W*: wild-type allele; *Δ*: deletion allele.

(B) Indicated cells were cultured on YPAD at 30°C for 1 day.
